# Adolescent mice exhibit lower reward sensitivity than adults

**DOI:** 10.3389/fnbeh.2025.1695375

**Published:** 2025-11-03

**Authors:** Klaudia Misiołek, Magdalena Chrószcz, Marta Klimczak, Aleksandra Rzeszut, Julia Netczuk, Barbara Ziółkowska, Łukasz Szumiec, Maria Kaczmarczyk-Jarosz, Zofia Harda, Jan Rodriguez Parkitna

**Affiliations:** ^1^Department of Molecular Neuropharmacology, Maj Institute of Pharmacology, Polish Academy of Sciences, Kraków, Poland; ^2^Department of Physiology, Maj Institute of Pharmacology, Polish Academy of Sciences, Kraków, Poland

**Keywords:** adolescence, reward sensitivity, conditioned place preference, social reward, cocaine reward, palatable food reward

## Abstract

**Introduction:**

Adolescence shapes adaptive adult behaviors. It is characterized by increased responsiveness to socially salient stimuli and heightened sensitivity to rewards in peer settings. The particular importance of social context during adolescence indicates that neural circuits responsible for social reward may develop along a different trajectory from those involved in non-social reward processing. However, this remains largely unexplored, as much of the existing research tends to focus on a single reward type, a specific age group of adolescents, or a single sex, thereby limiting a comprehensive understanding of how reward processing evolves across development.

**Methods:**

Here, we investigated how social, cocaine, and palatable food reward sensitivity is expressed in female and male C57BL/6 mice across early- (pubertal onset), mid- (peripubertal phase), and late- (sexual maturity) adolescence, compared to adults. We examined how these different rewards become associated with environmental contexts across developmental stages using the conditioned place preference (CPP) paradigm, a fundamental method for evaluating the motivational properties of stimuli.

**Results:**

We found that adolescent mice exhibited a lower preference for social and palatable food conditioned contexts, while cocaine CPP was not significantly affected by age. Comparisons across CPP tasks confirmed that age, rather than reward type or sex, was the primary factor influencing the magnitude of CPP. Overall, mid- and late-adolescent mice showed reduced mean CPP, with mid-adolescents exhibiting significantly lower odds of expressing a conditioned preference relative to adults.

**Discussion:**

These findings challenge the prevailing assumption that adolescent reward sensitivity universally enhances reward-context learning. Instead, we propose that the attenuated CPP observed in adolescence reflects lower reward sensitivity in emotionally neutral conditions, rather than deficits in associative learning or increased novelty seeking. Our results highlight how developmental stage influences reward-related behaviors and underscore the need for age- and sex-specific analyses in behavioral studies.

## Introduction

Adolescence is a crucial transitional phase into adulthood, marked by substantial physical, hormonal, and behavioral changes. It is characterized by heightened sensitivity to environmental influences and increased neural plasticity in brain regions involved in learning, motivation, emotional regulation, and social cognition (Spear, 2000; Knudsen, 2004; Dow-Edwards et al., 2019; Uhlhaas et al., 2023; Baker et al., 2025). While these developmental processes facilitate adaptive growth, they also create vulnerabilities to maladaptive changes, particularly under stress, which may trigger early onset of psychiatric disorders (Spear, 2000; Laviola et al., 2003; Laviola and Marco, 2011; Sheth et al., 2017). Central to these changes is the maturation of the brain’s reward system, which underlies increased reward-seeking behavior (Knudsen, 2004; Galván, 2010; Wahlstrom et al., 2010), novelty or sensation seeking (Adriani et al., 1998; Spear, 2000; Laviola et al., 2002; Vetter-O’Hagen and Spear, 2012), risk-taking (Laviola et al., 2003; de Water et al., 2014; Weisfeld and Shattuck, 2017), and susceptibility to drugs of abuse (Schramm-Sapyta et al., 2009; Arguello et al., 2024). It has been consistently reported that gender significantly influences reward-related behaviors (De Cock et al., 2016; Harden et al., 2018; van den Broek et al., 2020; Qutteina et al., 2021) as well as the onset, prevalence, and symptoms of mental disorders (Fairchild, 2011). Teenage girls and boys are more sensitive to social context, process social rewards differently than adults, and generally show heightened sensitivity to all rewards within peer settings (Spear, 2000, 2013; Doremus-Fitzwater et al., 2010). The subjective valuation of rewards undergoes significant changes across development (Csikszentmihalyi et al., 1977; Larson and Richards, 1991; Crone and Dahl, 2012); however, it remains unclear whether sensitivity to various rewarding stimuli (such as social, food, or drug) follows a common developmental pattern. Adolescents exhibit heightened neural responsiveness to socially salient stimuli, which coincides with increased risk-taking behaviors in social settings, including the initiation of substance use, thereby potentially increasing vulnerability to psychiatric disorders (Spear, 2000, 2013; Doremus-Fitzwater et al., 2010). The heightened importance of social cues in adolescence indicates that the development of neural circuits for social rewards may diverge from non-social rewards.

Adolescence and its associated behaviors show notable similarities between humans and laboratory rodents. The ages from 10 to 19 years in humans correspond to postnatal days 28–55 in mice, with both species experiencing similar developmental milestones, including the maturation of the brain’s reward system (Piekarski et al., 2017; Weisfeld and Shattuck, 2017). It has been reported that adolescent mice may have increased sensitivity to the rewarding effects of drugs of abuse (Burke and Miczek, 2014), especially under stress conditions (Kreibich et al., 2009; Schindler et al., 2012), and are less sensitive to their aversive effects (Tirelli et al., 2003; Schramm-Sapyta et al., 2009). Adolescent, but not adult mice, were observed to readily develop a preference for a social-conditioned context (Nardou et al., 2019) and demonstrate a stronger motivation to obtain highly palatable food (Friemel et al., 2010; Galván, 2013; Amancio-Belmont et al., 2017; Shteyn and Petrovich, 2025). These findings consistently support the idea that increased reward sensitivity during adolescence may strengthen associative memory, enhance the salience of related cues, and simultaneously facilitate the acquisition of both adaptive and maladaptive behaviors driven by environmental factors such as social experiences, drugs of abuse, or diet. Although this hypothesis is widely accepted, direct empirical evidence remains limited. In both humans and rodents, developmental processes are uneven and dynamic, with females and males maturing at different rates. Consequently, incorporating these factors into experiments is essential to capture and understand behavioral changes accurately. Nardou et al. (2019) demonstrated that conditioned place preference for social reward peaks during late adolescence compared to adulthood in both male and female mice. Additionally, a study on rats reported a sex-independent decline in social reward motivation during mid-adolescence, with both studies emphasizing the importance of multi-timepoint assessments throughout adolescence (Laviola et al., 2003; Tirelli et al., 2003; Murray et al., 2024). Equivalent analyses for other types of rewards are currently lacking. Moreover, despite the common view that adolescence is characterized by heightened reward sensitivity, it was historically debated whether a reduction in reward sensitivity could actually drive increased reward-seeking behaviors, potentially explaining vulnerability to drug abuse in humans (Spear, 2000).

The conditioned place preference (CPP) paradigm is a fundamental method for assessing the rewarding properties of a stimulus. Initially developed to evaluate the reinforcing effects of drugs (Rossi and Reid, 1976; Tzschentke, 1998; Bardo and Bevins, 2000), this approach has been adapted to examine various stimuli, including pain, food, and social cues. In this study, we used modified CPP tasks to measure reward-conditioned preference for social contact, palatable food, or cocaine in male and female C57BL/6 mice at three stages of adolescence: early (∼post-natal days 33, indicating puberty onset), mid (∼P38, peripubertal period), and late (∼P43, reflecting sexual maturity). Contrary to expectations, we observed that reward-conditioned preference was lower in mid-adolescents compared to adults, independent of reward type or sex. This finding challenges the simplistic notion that heightened reward sensitivity during adolescence directly leads to a strengthening of reward-context associations.

## Materials and methods

### Animals

Experiments were conducted on male and female C57BL/6 mice bred at the animal facility of the Maj Institute of Pharmacology, Polish Academy of Sciences in Krakow. The animals were housed at 22 ± 2 °C, with 40%–60% humidity, and maintained on a 12/12 h light/dark cycle (lights on at 7 AM). Mice had unlimited access to water and food (maintenance chow, 10 mm pellets, Altromin Spezialfutter, cat no. 1324, Germany). After weaning, they were housed in groups of 2–6 littermates per cage in standard Plexiglas cages (length 325 mm × width 170 mm) with wooden blocks for gnawing and nesting material. Female and male mice were kept in separate rooms. All behavioral procedures were performed during the light phase under dim light conditions (5–10 lux) with infrared lighting. Mice were handled for 3–5 consecutive days before each experiment by placing them in the experimenter’s hands for 2–3 min. Every 3 days, animals were marked on their tails for identification. Animals were moved to the experimental room at least 30 min prior to the experiments beginning. The age, sex, and weight of each animal at the start of the experiments are listed in Supplementary Table 1. All procedures were approved by the 2nd Local Bioethics Committee in Krakow (permit numbers 293/2020, 32/2021, 55/2024, 231/2024) and adhered to the guidelines of the European Parliament and the Council of 22 September 2010, on the protection of animals used for scientific purposes (Directive 2010/63/EU and Polish Law Dz.U. 2015 poz. 266). The experiments were planned and reported in accordance with ARRIVE guidelines (Percie du Sert et al., 2020).

### Social conditioned place preference

**FIGURE 1:**
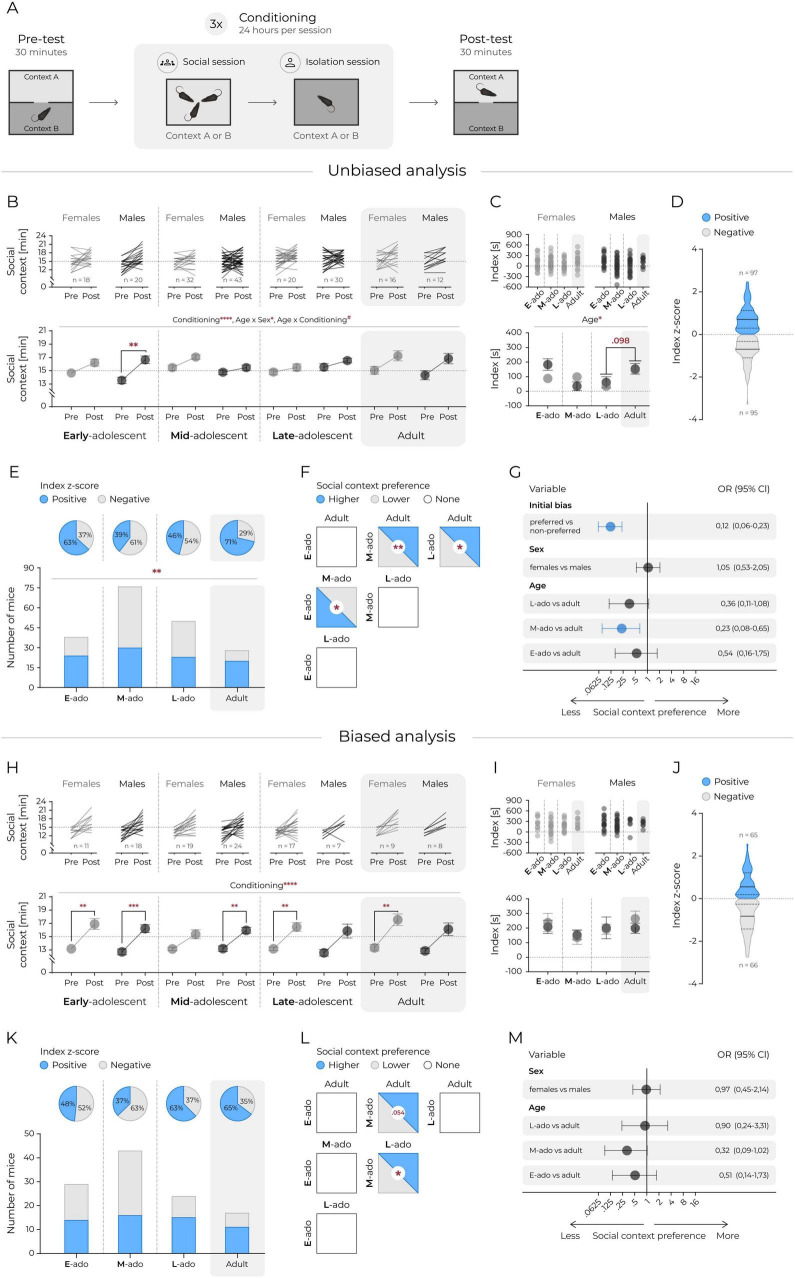
Adolescent mice show lower social context preference. **(A)** A schematic representation of the social CPP. **(B)** Time spent in the social context. Top panels: lines represent individual animals. Bottom panel: mean values. Circles and connecting lines represent means and matched values. Whiskers represent s.e.m. values. Dotted lines represent random value (i.e., 15 min). Females and males are shown in gray and black respectively. Statistical analysis was performed using 3-way ANOVA and *post hoc* Tukey’s HSD, **corresponds to *p* ≤ 0.01, ***p ≤ 0.001, ****p ≤ 0.0001. **(C)** Difference in seconds between time spent in social context posttest and time spent in social context pretest (index). Top panels: individual animals. Bottom panel: mean values. Whiskers represent s.e.m. values. Dotted lines represent no change. Statistical analysis was performed using 2-way ANOVA and *post hoc* Tukey’s HSD, *corresponds to *p* ≤ 0.05. **(D)** Distribution of social context preference in the posttest, categorized relative to the group mean (all animals). Sex and age data were combined. Index z-score calculated as: (index of individual animal – mean index of all mice)/standard deviation of index of all mice). Context preference interpreted according to index z-score value: positive, i.e., >0 or negative, i.e., <0. Solid line represents the median, dashed lines mark the quartiles. **(E)** Fractions of animals exhibiting positive (i.e., >0) or negative (i.e., <0) index z-score values are shown. Top panels (pie charts): percent of animals expressing positive (blue) or negative (white) index z-score in each age group. Bottom panels: proportion of animals expressing positive (blue bars) or negative (white bars) index z-score. χ^2^ test revealed no significant differences between proportions. **(F)** Pairwise comparisons of social context preference based on the proportion of animals with positive (i.e., >0) and negative (i.e., <0) index z-score values. Each square represents a comparison between each age group. The fill color indicates the predominance of animals expressing higher (blue), lower (gray) social context preference, or no difference (white) between groups. All pairwise χ^2^ comparisons were performed, *corresponds to *p* ≤ 0.05, **corresponds to *p* ≤ 0.01. **(G)** Logistic regression estimates of the effects of initial bias, sex and age on the odds of increased preference for social context based on the index z-score. The circles and horizontal lines indicate the odds ratio and corresponding 95% confidence intervals. Statistically significant effects are marked blue. Panels **(H–M)** correspond to **(B–G)** but with analyses performed only on animals initially preferring the isolation context, i.e., the equivalent of a biased experimental design. **(H)** Time spent in the social context. Top panels: lines represent individual animals. Bottom panel: mean values. Whiskers represent s.e.m. values. Dotted lines represent random value (i.e., 15 min). Females and males are shown in gray and black respectively. Statistical analysis was performed using 3-way ANOVA and *post hoc* Tukey’s HSD, **corresponds to *p* ≤ 0.01, ***p ≤ 0.001, ****p ≤ 0.0001. **(I)** Difference in seconds between time spent in social context posttest and time spent in social context pretest (index). Top panels: individual animals. Bottom panel: mean values. Circles and connecting lines represent means and matched values. Whiskers represent s.e.m. values. Dotted lines represent no change. Statistical analysis was performed using 2-way ANOVA revealed no significant differences between groups. **(J)** Distribution of social context preference in the posttest, categorized relative to the group mean (all animals). Sex and age data were combined. Index z-score calculated as: (index of individual animal – mean index of all mice)/standard deviation of index of all mice). Context preference interpreted according to index z-score value: positive, i.e., >0 or negative, i.e., <0. Solid line represents the median, dashed lines mark the quartiles. **(K)** Fractions of animals exhibiting positive (i.e., >0) or negative (i.e., <0) index z-score values are shown. Top panels (pie charts): percent of animals expressing positive (blue) or negative (white) index z-score in each age group. Bottom panels: proportion of animals expressing positive (blue bars) or negative (white bars) index z-score. χ^2^ test revealed no significant differences between proportions. **(L)** Pairwise comparisons of social context preference based on the proportion of animals with positive (i.e., >0) and negative (i.e., <0) index z-score values. Each square represents a comparison between each age group. The fill color indicates the predominance of animals expressing higher (blue), lower (gray) social context preference, or no difference (white) between groups. All pairwise χ^2^ comparisons were performed, *corresponds to *p* ≤ 0.05. **(M)** Logistic regression estimates of the effects of sex and age on the odds of increased preference for social context based on index z-score. The circles and horizontal lines indicate the odds ratio and corresponding 95% confidence intervals. There were no statistically significant effects, with one result approaching statistical significance adult vs. M-ado, *p* = 0.059.

Social CPP was performed as described before (Harda et al., 2022, 2025a,b; Misiołek et al., 2023) and is a revised version of the protocol by Panksepp and Lahvis (2007), with modifications introduced by Dölen et al. (2013), Nardou et al. (2019). Compared to the procedure we described previously, the main difference are the types of beddings. Animals were housed on corn bedding prior to experiments (corn, 2 mm, Rehofix MK2000, Germany). The test was conducted in a custom-made two-chamber apparatus with compartments differing in bedding type (context A: aspen, ABEDD, Latvia or Tapvei GLP, Estonia, context B: 1/8′ Pelleted Cellulose, Scott Pharma Solutions, cat no. L0107, USA) and wooden blocks (context A: cuboid, context B: cube, both from Zoolab, Poland). The social CPP involved three phases: pre-test, conditioning, and post-test (Figure 1A). Behavior during the pre- and post-tests was recorded using a camera (acA1300 – 60 gm, Basler, Germany). During pre-test, mice freely explored the two-chamber cages for 30 min. Mice that spent more than 70% of the time in any context were excluded. To counterbalance contextual bias, half of the animals were assigned to context A as the social-paired environment, while the other half were assigned to context B. The conditioning phase consisted of six consecutive sessions. During the social conditioning session, mice and their littermates were moved to new home cages with one of the conditioning contexts for 24 h. During the isolation session, mice were placed separately in new home cages with the other conditioning context for 24 h. Conditioning started with a social session, and the context alternated daily (social-isolation-social-isolation-social-isolation). The post-test was conducted the day after the last conditioning session. Behavior was automatically analyzed using EthoVision XT 15 software (Noldus, Netherlands). The social place preference index was calculated by subtracting the time spent in the social context during the pre-test from the time spent in the social context during the post-test.

### Cocaine conditioned place preference

**FIGURE 2:**
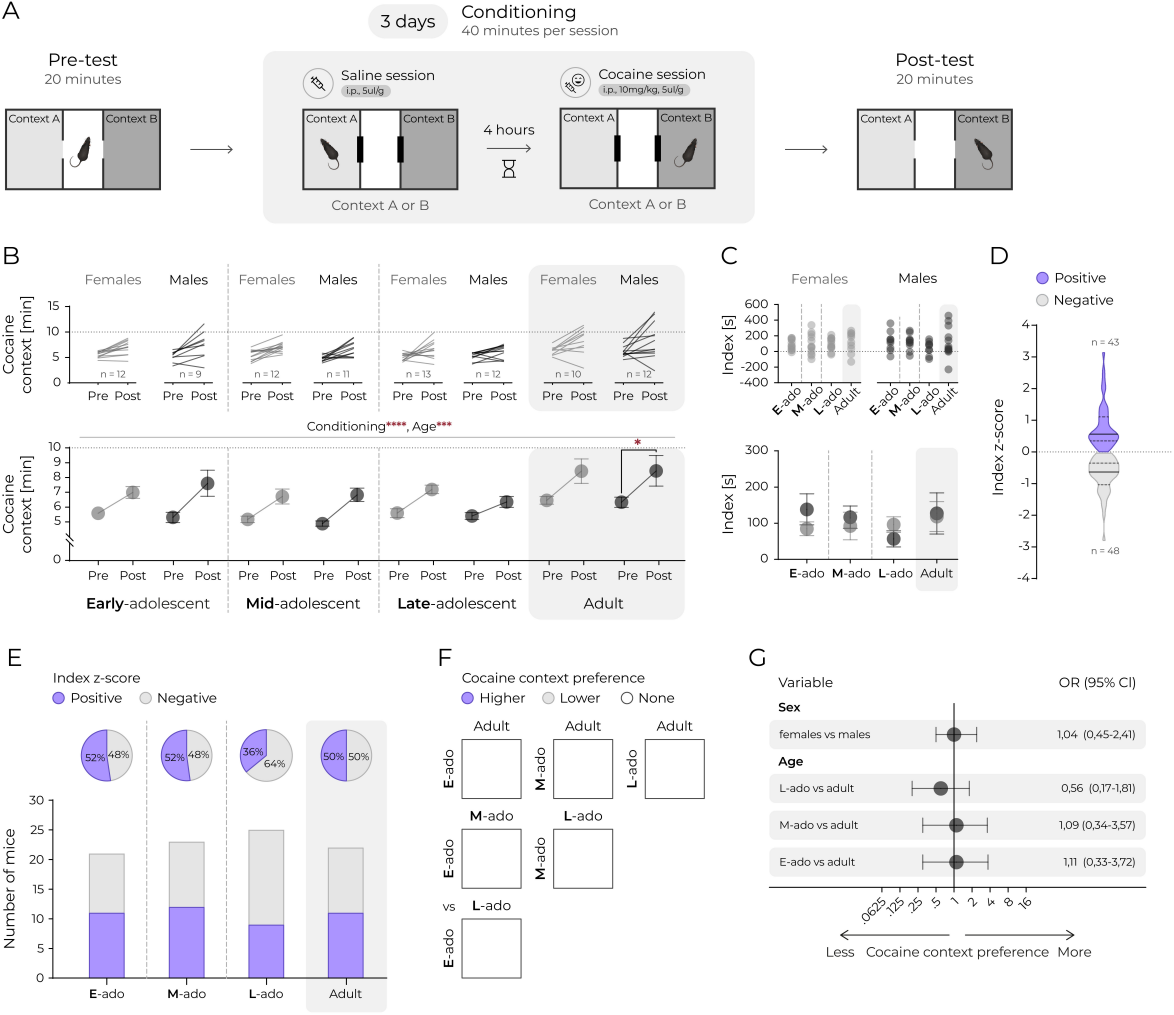
Age has no significant effect on cocaine conditioned place preference. **(A)** A schematic representation of the cocaine CPP. **(B)** Time spent in the cocaine context. Top panels: lines represent individual animals. Bottom panel: mean values. Circles and connecting lines represent means and matched values. Whiskers represent s.e.m. values. Dotted lines represent random value (i.e., 10 min). Females and males are shown in gray and black, respectively. Statistical analysis was performed using 3-way ANOVA and *post hoc* Tukey’s HSD, ***corresponds to *p* ≤ 0.001, ****p ≤ 0.0001. **(C)** Difference in seconds between time spent in cocaine context posttest and time spent in saline context pretest (index). Top panels: individual animals. Bottom panel: mean values. Whiskers represent s.e.m. values. Dotted lines represent no change. Statistical analysis was performed using 2-way ANOVA and revealed no significant differences between groups. **(D)** Distribution of cocaine context preference in the posttest, categorized relative to the group mean (all animals). Sex and age data were combined. Index z-score calculated as: (index of individual animal – mean index of all mice)/standard deviation of index of all mice. Context preference interpreted according to index z-score value: positive, i.e., >0 or negative, i.e., <0. The solid line represents the median, and the dashed lines mark the quartiles. **(E)** Fractions of animals exhibiting positive (i.e., >0) or negative (i.e., <0) index z-score values are shown. Top panels (pie charts): percent of animals expressing positive (purple) or negative (white) index z-score in each age group. Bottom panels: proportion of animals expressing positive (purple bars) or negative (white bars) index z-score. χ^2^ test revealed no significant differences between proportions. **(F)** Pairwise comparisons of cocaine context preference based on the proportion of animals with positive (i.e., >0) and negative (i.e., <0) index z-score values. Each square represents a comparison between each age group. The fill color indicates the predominance of animals expressing higher (purple), lower (gray) cocaine context preference, or no difference (white) between groups. All pairwise χ^2^ comparisons were not significant. **(G)** Logistic regression estimates of the effects of sex and age on the odds of increased preference for social context based on index z-score. The circles and horizontal lines indicate the odds ratio and corresponding 95% confidence intervals. There were no statistically significant effects.

The cocaine CPP test was conducted using an automatic three-chamber apparatus (Med Associates, St. Albans, VT, USA, MED-CPP-MSAT) as described previously (Sora et al., 1998; Harda et al., 2020, 2025b). The apparatus features two compartments that differ in color and tactile cues, along with a middle compartment that has guillotine doors separating them. Photobeams automatically tracked movement and time spent in each chamber. The CPP paradigm consisted of three phases: pre-test, conditioning, and post-test (Figure 2A). On the first day, the pre-test was conducted to determine the initial preference. Mice were placed in the middle compartment and allowed to explore the entire apparatus for 20 min. Mice that spent more than 70% of their time in one of the conditioning chambers, excluding time in the middle chamber, were excluded. Conditioning sessions took place over the next three days. Each conditioning day included two 40-min sessions: one with saline, and after about 4 h in home cages, a cocaine session (saline-cocaine, saline-cocaine, saline-cocaine). The design was biased; the less preferred chamber was paired with the cocaine injection (cocaine hydrochloride dissolved in saline, i.p., 10 mg/kg, 5 μl/g, cocaine hydrochloride, Toronto Research Chemicals; TRC, Toronto, North York, ON, Canada), while the more preferred chamber was paired with the saline injection (i.p., 5 μl/g, Polpharma, Poland). The post-test was performed in the same way as the pre-test, the day after the last conditioning session. The place preference index was calculated by subtracting the time spent in the cocaine-paired chamber during the pre-test from the time spent in the cocaine-paired chamber during the post-test.

### Palatable food conditioned place preference

**FIGURE 3:**
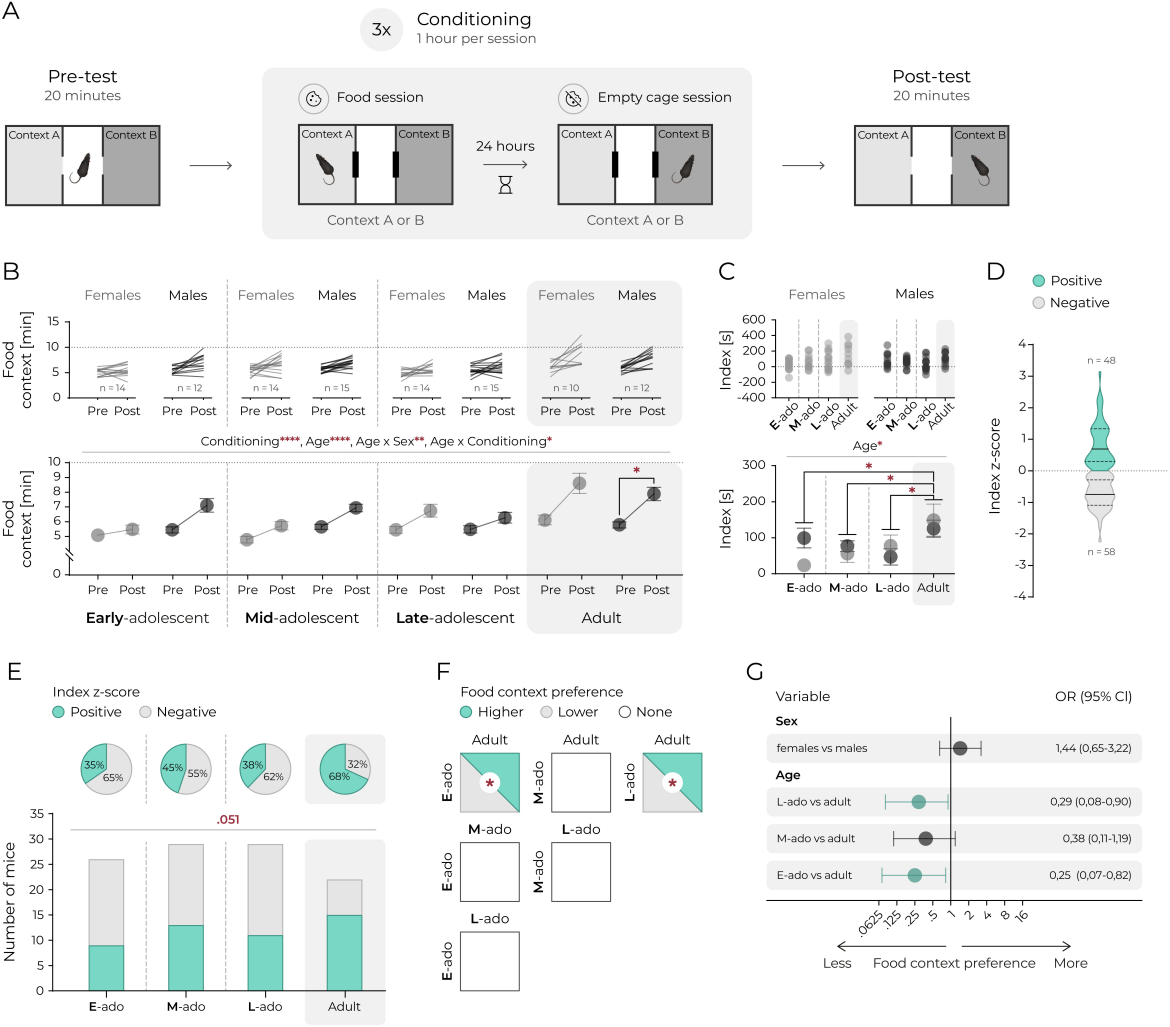
Palatable food preference is lower in adolescent mice. **(A)** A schematic representation of the food CPP. **(B)** Time spent in the food context. Top panels: lines represent individual animals. Bottom panel: mean values. Circles and connecting lines represent means and matched values. Whiskers represent s.e.m. values. Dotted lines represent random value (i.e., 10 min). Females and males are shown in gray and black respectively. Statistical analysis was performed using 3-way ANOVA and *post hoc* Tukey’s HSD, *corresponds to *p* ≤ 0.05, **, *p* ≤ 0.01, ****p ≤ 0.0001. **(C)** Difference in seconds between time spent in food context posttest and time spent in empty context pretest (index). Top panels: individual animals. Bottom panel: mean values. Whiskers represent s.e.m. values. Dotted lines represent no change. Statistical analysis was performed using 2-way ANOVA and *post hoc* Tukey’s HSD, *corresponds to *p* ≤ 0.05. **(D)** Distribution of food context preference in the posttest, categorized relative to the group mean (all animals). Sex and age data were combined. Index z-score calculated as: (index of individual animal – mean index of all mice)/standard deviation of index of all mice). Context preference interpreted according to index z-score value: positive, i.e., >0 or negative, i.e., <0. Solid line represents the median, dashed lines mark the quartiles. **(E)** Fractions of animals exhibiting positive (i.e., >0) or negative (i.e., <0) index z-score values are shown. Top panels (pie charts): percent of animals expressing positive (green) or negative (white) index z-score in each age group. Bottom panels: proportion of animals expressing positive (green bars) or negative (white bars) index z-score. χ^2^ test indicated a difference between proportions approaching statistical significance, *p* = 0.051. **(F)** Pairwise comparisons of food context preference based on the proportion of animals with positive (i.e., >0) and negative (i.e., <0) index z-score values. Each square represents a comparison between each age group. The fill color indicates the predominance of animals expressing higher (green), lower (gray) food context preference, or no difference (white) between groups. All pairwise χ^2^ comparisons were performed, *corresponds to *p* ≤ 0.05. **(G)** Logistic regression estimates of the effects of sex and age on the odds of increased preference for social context based on index z-score. The circles and horizontal lines indicate the odds ratio and corresponding 95% confidence intervals. Statistically significant effects are marked green.

A palatable food CPP test was conducted in the same experimental setup as the cocaine CPP, using a protocol adapted from Clough et al. (2018). Mice were housed individually and habituated to a palatable food mixture placed in their home cage on a flat glass plate. Each day, they received a fresh portion consisting of 2 pieces of regular chow, 1/3 of an open Oreo cookie (cream side up), 2 Cheetos, and 5 Froot Loops; the nutritional details of these foods are listed in Supplementary Table 2. This habituation process was conducted over two consecutive days prior to the experiment. On the pre-test day, mice were grouped by sex with their littermates in their original home cages (without palatable food, but with chow and water available freely) and allowed to habituate in the experimental room. The pre-test was identical to the cocaine CPP test procedure (Figure 3A). Mice that spent over 70% of their time (excluding time in the middle box) in any conditioning box were excluded from the analysis. An initial bias test was performed, and a biased design was used to assign the less preferred context as the palatable food context (with the food mix) and the more preferred one as the empty cage (without food). Conditioning sessions lasted six consecutive days, with one 60-min session each day. To motivate food-seeking behavior, standard chow was removed from the cages 2 h before and 2 h after each session. Sessions alternated daily, starting with the palatable food session, followed by the empty cage session the next day, and so on (food-empty-food-empty-food-empty). The post-test was then performed, and the place preference index was calculated as in the cocaine CPP test.

### Data analysis

All statistical analyses were performed using GraphPad Prism 10.4.2 for Windows (GraphPad Software, n.d.), except for four-way analysis of variance (ANOVA), which was conducted in R (4.5.0) (R Development Core Team, 2025). Animals excluded from experiments based on pre-test criteria (those that spent more than 70% of time in any conditioning context) are listed in Supplementary Table 1. No outliers were detected in the experimental data using the ROUT test. The results were analyzed using ANOVA followed by Tukey’s HSD *post hoc* test. Additionally, χ2 was used to analyze categorical data, and logistic regression was used to assess the effects of initial bias, sex, age, and reward type on the preference to the conditioned context as a binary outcome. Correlations were evaluated using linear regression. Statistical tests and sample sizes are included in the main text or in figure legends.

## Results

### Adolescent mice show lower social context preference

We first investigated the rewarding effects of social contact using the social CPP test (Figure 1A), in which interaction with a same sex sibling or social isolation was paired with two distinct novel bedding types. An increase in time spent in the social context from pre- to post-test was interpreted as evidence of the rewarding effects of social contact. Across all groups, mice showed a significant increase in time spent in the social context following conditioning (Figure 1B, 3-way ANOVA: Fconditioning_(1_._184)_ = 46.53, *p* < 0.0001). While no main effect of age or sex was detected (Fage_(3_._184)_ = 0.67, *p* = 0.555, Fsex_(1_._184)_ = 1.10, *p* = 0.295) a significant age and sex interaction emerged (Fage*sex_(3_._184)_ = 3.13, *p* = 0.027; Fage*conditioning_(3_._184)_ = 2.54, *p* = 0.058; Fsex*conditioning_(1_._184)_ = 0.420, *p* = 0.518; Fage*sex*conditioning_(3_._184)_ = 1.31, *p* = 0.273), indicating potential age-dependent differences between males and females. To focus specifically on the effect of the conditioning stimulus and control for initial context preference, we next analyzed the difference in time spent in the social-paired context between post-test and pre-test (i.e., the social preference index; Figure 1C). The advantage of the index over the preference during the post-test (i.e., score) is that it is not confounded by animals that have a consistent preference for the conditioned compartment in both pre- and post-test. We found a significant effect of age (Figure 1C, 2-way ANOVA: Fage_(3_._184)_ = 2.97, *p* = 0.033), but no significant effect of sex or age and sex interaction (Fsex_(1_._184)_ = 0.34, *p* = 0.561; Fage*sex_(3_._184)_ = 1.66, *p* = 0.179). While *post hoc* comparisons did not reach statistical significance, a trend toward lower social preference in late-adolescents compared to adults was observed. Given the absence of sex effects, we simplified subsequent analyses by ignoring the sex effect and converting the CPP result to a binary outcome based on the sign of the z-score (i.e., above or below the mean of all cases) (Figure 1D). Cumulative frequency distributions revealed a significant overall difference in social context preference across age groups (Figure 1E, χ^2^_(3)_ = 11.44, *p* = 0.009). Specifically, mid- and late-adolescent mice showed a significantly lower proportion of animals exhibiting social CPP compared to adults (Figures 1E, F, 39% vs. 71%, χ^2^_(1)_ = 8.37, *p* = 0.004; 46% vs. 71%, χ^2^_(1)_ = 4.69, *p* = 0.030, respectively). Additionally, mid-adolescents showed a significantly lower proportion of social preference compared to early adolescents (Figures 1E, F, 39% vs. 63%, χ^2^_(1)_ = 5.70, p = 0.017). To account for potential confounding factors, we conducted a logistic regression analysis that included age, sex, and initial preference bias as predictors of binary CPP expression (Figure 1G). This analysis confirmed that mid-adolescent mice had significantly reduced odds of developing a social context preference relative to adults (95% CI [0.08, 0.65], *p* = 0.007). Importantly, initial preference had a strong influence on CPP expression, with a significant effect observed on the odds of showing a preference post-conditioning (odds ratio 95% CI [0.06, 0.23], *p* < 0.0001).

However, analysis of the data revealed that changes in CPP were confounded by the type of bedding used during conditioning. Although the full dataset showed no significant group-level preference for either context before conditioning (cellulose preference 49.95 ± 9%, one-sample *t*-test vs. 50%, *p* > 0.05), age and sex influenced initial context preferences when examined separately. Specifically, a significant effects of sex and an age × sex interaction on baseline preference was observed (Supplementary Figure 1A, 2-way ANOVA Fage_(3_._21)_ = 1. 46, *p* = 0.230, Fsex_(1_._21)_ = 10.82, *p* = 0.001, Fage*sex_(3_._21)_ = 3.89, *p* = 0.009). In line with this, the proportion of animals developing a conditioned preference differed across groups: males were more likely than females to exhibit CPP (Supplementary Figure 1B, 95% CI [1.30, 4.10], *p* = 0.005), and late-adolescents were less likely than adults (95% CI [0.14, 0.97], *p* = 0.044). Logistic regression analysis confirmed that mice that had an initial preference for the social context had significantly higher preference for it in the post-test compared to animals with an initial preference for the isolate context (Figure 1G, 95% CI [0.06, 0.23], *p* < 0.0001). However, conditioning effects (i.e., increase in preference for stimulus-paired context) were only observed in the animals for whom the social interaction was paired with their initially less-preferred context (Supplementary Figure 2A, 4-way ANOVA significant effects: Finitialbias_(4_._03)_ = 95.0, *p* < 0.001, Fconditioning_(3_._36)_ = 52.7, *p* < 0.001, Fconditioning*initialbias_(3_._36)_ = 59.4, *p* < 0.001, Fsex*age_(4_._03)_ = 3.90, *p* < 0.01, Fsex*age*conditioning_(3_._36)_ = 2.66, *p* < 0.01). Importantly, there was no significant correlation between pre- and post-test time in the social-paired context (r^2^ = 0.02, *p* > 0.05), suggesting that CPP outcomes were not simply driven by pre-existing preferences. These results reinforce previous findings highlighting the confounding role of reward-independent context biases in CPP paradigms (Tzschentke, 1998; Bardo and Bevins, 2000; Cunningham et al., 2003; Ávila Gámiz et al., 2020; Harda et al., 2022), and contribute to the ongoing debate regarding the use of biased versus unbiased designs (Tzschentke, 1998, 2007; Cunningham et al., 2003; Ávila Gámiz et al., 2020). Therefore, to ensure comparability with the design of cocaine and palatable CPP tests, we restricted subsequent analyses to animals exhibiting no initial preference for the social context.

When the analysis was performed only on animals that had initial preference for the isolate context, we have again found a significant increase in time spent in the social context from pre- to post-test, with no effects of sex, age, or their interactions (Figure 1H, 3-way ANOVA: Fconditioning_(1_._105)_ = 111.6, *p* < 0.0001; Fage_(3_._105)_ = 0.65, *p* = 0.585; Fsex_(1_._105)_ = 2.10, *p* = 0.150; Fage*sex_(3_._105)_ = 0.991, *p* = 0.400; Fage*conditioning_(3_._105)_ = 1.14, *p* = 0.337; Fsex* conditioning_(1_._105)_ = 0.072, *p* = 0.788; Fage*sex*conditioning_(3_._105)_ = 0.273, *p* = 0.845). This observation is consistent with the results of the unbiased analysis of the complete set of data. Likewise, there were no significant effects of sex or age on the social preference index, which replicates the observation from the analysis of the complete set (Figure 1I, 2-way ANOVA: Fage_(3_._104)_ = 1.58, *p* = 0.199; Fsex_(1_._104)_ = 0.22, *p* = 0.638; Fage*sex_(3_._104)_ = 0.28, *p* = 0.841). As previously, to simplify further analyses, the effect of sex was excluded, and preference was binarized based on z-score (Figure 1J). Cumulative frequency distributions showed no significant differences in social context preference among age groups (Figure 1K, χ^2^ test, *p* > 0.05). Still, a significantly higher proportion of late-adolescent mice preferred the social context compared to mid-adolescents in a pairwise comparison (Figure 1L, 63% vs. 37%, χ^2^_(1)_ = 3.96, *p* = 0.046). Logistic regression did not reveal significant effects; the relative odds between adult vs. mid-adolescent mice did not reach significance (Figure 1M, *p* = 0.059). Overall, these findings are consistent with those from the full dataset, although the reduced sample size limited statistical power. Together, both unbiased and biased analyses suggest two separate effects: a lower preference for social-conditioned context in adolescent mice compared to adults, possibly varying across adolescent stages, and a qualitative developmental difference in the proportion of animals developing a preference for the context associated with social contact.

### Age has no significant effect on cocaine conditioned place preference

Next, we investigated whether the reduction in social CPP observed during adolescence reflects a social-specific phenomenon or a broader attenuation of reward learning. First, we assessed cocaine-induced CPP, as this paradigm is well-established and cocaine is a drug of abuse known to produce robust CPP with relatively few confounding or aversive effects. Cocaine reward was tested using a three-compartment CPP test with a biased design (Figure 2A). As expected, mice exhibited a significant increase in time spent in the cocaine-paired context (Figure 2B, 3-way ANOVA: Fconditioning_(1_._83)_ = 67.53, *p* < 0.0001, Fage_(3_._83)_ = 6.26, *p* = 0.001, Fsex_(1_._83)_ = 0.21, *p* = 0.648, Fage*sex_(3_._83)_ = 0.309, *p* = 0.819, Fage*conditioning_(3_._83)_ = 0.64, *p* = 0.590, Fsex*conditioning_(1_._83)_ = 0.218, *p* = 0.642, Fage*sex*conditioning_(3_._83)_ = 0.61, *p* = 0.614). Although a significant effect of age was detected on the increase in time spent in cocaine-paired context, this effect was not observed when the preference was analyzed using a preference index (Figure 2C, 2-way ANOVA: Fage_(3_._83)_ = 0.64, *p* = 0.590, Fsex_(1_._83)_ = 0.22, *p* = 0.642, Fage*sex_(3_._83)_ = 0.61, *p* = 0.614). Further, when cocaine reward preference was treated as a binary outcome (Figure 2D), comparable proportions of mice across developmental stages exhibited cocaine CPP. Statistical analysis confirmed no significant differences in preference proportions among age groups (Figure 2E, χ^2^ test, *p* > 0.05) or in pairwise comparisons (Figure 2F, all χ^2^ test, *p* > 0.05). Logistic regression analysis similarly revealed no significant effects of age or sex on cocaine preference (Figure 2G).

### Palatable food preference is lower in adolescent mice

To determine whether adolescent reduction in social, but not cocaine CPP, reflects a phenomenon specific to social reward or extends to other natural rewards, we next evaluated CPP using palatable food in a setup similar to cocaine CPP (Figure 3A). This method followed the procedure described by Clough et al. (2018), but without extended chow restriction. Palatable food produced a significant increase in preference for the associated context, with significant interactions between age and sex, and between age and conditioning (Figure 3B, 3-way ANOVA: Fconditioning_(1_._98)_ = 81.01, *p* < 0.0001, Fage_(3_._98)_ = 12.08, *p* < 0.0001, Fsex_(1_._98)_ = 3.52, *p* = 0.064, Fage* sex_(3_._98)_ = 5.49, *p* = 0.002, Fage*conditioning_(3_._98)_ = 3.65, *p* = 0.015, Fsex*cond_(1_._98)_ = 0.34, *p* = 0.562, Fage*sex*cond_(3_._98)_ = 1.82, *p* = 0.149). Analysis of preference using index revealed a significant effect of age but no effects of sex or age and sex interaction (Figure 3C, 2-way ANOVA: Fage_(3_._98)_ = 3.65, *p* = 0.015, Fsex_(1_._98)_ = 0.34, *p* = 0.642, Fage*sex_(3_._88)_ = 1.82, *p* = 0.149). Early-, mid-, and late-adolescent mice exhibited significantly lower conditioned preference compared to adults (Figure 3C). When preference, excluding sex as a factor, was categorized as a binary outcome (Figure 3D), cumulative frequency analysis showed a trend toward an age effect (Figure 3E, χ^2^_(1)_ = 3.795, *p* = 0.051). Pairwise comparisons revealed significantly lower proportions of early- or late-adolescent mice expressing food reward preference relative to adults (Figure 3F, 35% vs. 68%, χ^2^_(1)_ = 8.37, *p* = 0.004, 38% vs. 68%, χ^2^_(1)_ = 4.69, *p* = 0.030). These differences remained significant in logistic regression analyses (Figure 3G), with odds of preferring the food context were lower by 25% for early-adolescents (95% CI [0.07, 0.82], *p* = 0.026) and by 28% for late-adolescent mice (95% CI [0.08, 0.90], *p* = 0.036) relative to adults. Pre-test time spent in the reward context positively correlated with post-test time for both cocaine (r^2^ = 0.17, *p* < 0.0001) and palatable food CPP (r^2^ = 0.10, *p* = 0.001). No evidence suggested that differences in motor activity confounded the results for any reward CPP (Supplementary Figures 3A–D).

### Adolescent mice show decreased reward place preference compared to adults

**FIGURE 4:**
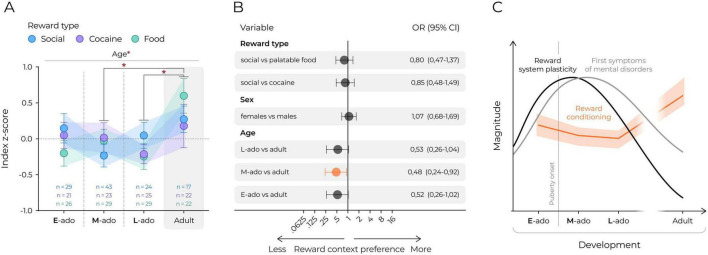
Adolescent mice show decreased reward place preference compared to adults. **(A)** Comparison of relative conditioned context preference between social, cocaine and food reward. Sex data were combined. Index z-score calculated for each CPP experiment separately as: (index of individual animal – mean index of all mice)/standard deviation of index of all mice). The colored circles represent mean values, the color-corresponding shadows and whiskers represent s.e.m. values. Statistical analysis was performed using 2-way ANOVA and *post hoc* Tukey’s HSD, *corresponds to *p* ≤ 0.05. **(B)** Logistic regression estimates of the effects of reward type, sex and age on the odds of increased preference for reward context based on logistic regression. The circles and horizontal lines indicate the odds ratio and corresponding 95% confidence intervals. Statistically significant effects are marked orange. **(C)** A graphical representation of the developmental trajectories of social and non-social conditioned place preference in mice. The orange curve with the color-corresponding shadow represent mean and s.e.m. of reward conditioning (based on Figure 4A). The black and gray lines indicate the sensitive periods for reward system and emergence of first symptoms of mental disorders in humans, respectively [data based on (Uhlhaas et al., 2023)].

Lastly, we performed a direct comparison of age effects on conditioned preference across all reward types by analyzing normalized z-scores of the preference indices. This approach controlled for variability in the post-test durations and excluded sex as a factor to reduce analytical complexity. There was a significant main effect of age on conditioned preference, but no effects of reward type and no interaction between age and reward type (Figure 4A, 2-way ANOVA: Freward_(2_._30)_ = 0.064, *p* = 0.938, Fage_(3_._30)_ = 3.21, *p* = 0.023, Freward*age_(6_._30)_ = 1.07, *p* = 0.381). Specifically, adult mice showed higher reward CPP compared to mid- and late-adolescents (Figure 4A). In line with these results, logistic regression analysis confirmed a robust age effect as the odds of expressing conditioned preference were decreased by 48% for mid-adolescent (Figure 4B, 95% CI [0.24, 0.92], *p* = 0.028) relative to adult mice, given that the other variables in the model are held constant. No significant main effects of sex or reward type on the conditioned reward preference were identified. To contextualize these developmental changes, Figure 4C presents reward CPP trajectories in relation to puberty onset, the maturation timeline of the reward system, and the period of increased vulnerability to mental disorders, based on Uhlhaas et al. (2023). Although speculative, this comparison suggests that the decrease in CPP observed during mid-adolescence coincides with a period of major neurodevelopmental transition.

## Discussion

We found that adolescent mice exhibited weaker reward-conditioned place preference compared to adults, a pattern that was largely consistent across reward types and independent of sex. This result is intuitively at odds with the commonly reported increase in reward sensitivity during adolescence (Spear, 2000, 2013) and in conflict with findings showing robust social CPP in adolescent mice (Nardou et al., 2019).

Conditioned place preference is a well-established method for evaluating the rewarding effects of stimuli; however, outcomes are susceptible to methodological variables, including setup and behavioral parameters, which can significantly influence results (Bardo and Bevins, 2000; Cunningham et al., 2003; Ávila Gámiz et al., 2020; Yates, 2023). One critical factor is initial context bias, which can substantially shape CPP expression (Tzschentke, 1998; Cunningham et al., 2003). Although an unbiased design allows for the detection of both appetitive and aversive responses, the strength of CPP can vary substantially across stimuli (Kummer et al., 2014), and even subtle contextual preferences may obscure reward-driven learning. To address this, we followed task-specific designs, an unbiased design for social CPP (Harda et al., 2022, 2025a; Misiołek et al., 2023), a biased design for cocaine CPP (Harda et al., 2020), and adhered to the original, biased protocol for palatable food CPP (Clough et al., 2018). To account for potential bias-related effects, we analyzed both biased and unbiased versions of social CPP and found consistent results. As emphasized by Yates (2023), the interpretation of CPP depends critically on both experimental design and the choice of behavioral metrics, typically the index (change from pre- to post-test) or the score (post-test difference in preference between compartments). In our study, animals that already exhibited a strong initial preference for the social context showed no further increase following conditioning, and all animals preferred the social context at post-test, irrespective of initial bias, suggesting potential ceiling effects. Analyses conducted on both the full dataset and the biased subset of social CPP data yield consistent results: the effects of age and no effect of sex, although the specific behavioral metrics reaching statistical significance varied between analyses. Finally, we have to emphasize that our study was not designed to directly compare the sensitivity to social versus non-social rewards. Evaluation of differences in motivational value between reward types requires concurrent conditioning paradigms and assessment of how preferences shift as a function of parameters such as dose [e.g., (Kummer et al., 2014)]. We cannot exclude the possibility that different doses of cocaine might have produced different magnitudes of CPP, or that longer periods of restricted food access could have enhanced the motivational salience of palatable food reward. In our protocol, food deprivation was deliberately minimized to reduce such confounds. Moreover, while social CPP was based on a two-chamber design, palatable food and cocaine CPP used a three-chamber apparatus, which precludes direct comparison of times spent in the stimulus-paired context. For these reasons, we focused not on the absolute magnitude of CPP between reward types, but rather on the developmental trajectories and the influence of sex and age, the factors that are not inherently test-type dependent.

Our social CPP findings align with recent work by Murray et al. (2024), who reported a decrease in social reward motivation in mid-adolescent rats using operant conditioning, although with the highest motivation observed in early adolescents. In contrast, Nardou et al. (2019) have elegantly demonstrated in the social CPP task that social contact is rewarding in male and female adolescent but not adult mice, a result we have previously replicated (Harda et al., 2022). However, when we extended conditioning and used siblings as social partners, we observed robust social CPP in 14-week-old adult mice. This aligns with the role of social context, prior social experiences, internal state, and kinship in social CPP (Panksepp and Lahvis, 2007; Cann et al., 2020; Harda et al., 2022; Misiołek et al., 2023). In humans, adolescents show transient increases in social motivation toward non-kin peers rather than family members (Spear, 2000, 2013), which may explain why social CPP could be enhanced during adolescence when conditioned with unfamiliar conspecifics. In the present study, we found no consistent sex differences in social CPP, in line with several studies (Panksepp and Lahvis, 2007; Nardou et al., 2019; Murray et al., 2024), though others reported higher social CPP in male rats compared to females (Douglas et al., 2004; Grotewold et al., 2014) or higher social CPP in female mice compared to males (Cann et al., 2020), depending on housing and prior social isolation.

For palatable food CPP, our findings contrast with multiple reports showing enhanced food intake and motivation during adolescence (Friemel et al., 2010; Galván, 2013; Amancio-Belmont et al., 2017; Shteyn and Petrovich, 2025), especially during mid-puberty (Friemel et al., 2010). Shteyn and Petrovich (2025), for example, found that adult rats prefer palatable food regardless of satiety, whereas adolescents do so only when satiated. Moreover, prior studies showed that adult mice form CPP for high-fat/sugar food mixes but not for chow alone (Clough et al., 2018), suggesting that the nutritional composition, rather than caloric content *per se*, drives food reward. In our study, we imposed a mild (2-h) food deprivation prior to conditioning, aiming to enhance motivation while minimizing stress. However, this protocol may have differentially affected motivation: adults may have selectively consumed more rewarding components, strengthening context-reward associations, whereas adolescents may have consumed food more evenly, leading to weaker CPP. Food consumption was not directly monitored, which limits this interpretation. As with social CPP, we found no consistent sex effects. Apparent differences between early-adolescent males and females were attributable to increased time spent in the neutral compartment by females, likely reflecting anxiety-like behavior. This lack of robust sex effects aligns with previous findings (Anderson and Petrovich, 2015, 2018), though others have reported stronger food CPP in females (Sinclair et al., 2017; Shteyn and Petrovich, 2025; Shteyn et al., 2025).

Adolescent drug-related reward sensitivity is often linked to heightened stress responsivity (Burke and Miczek, 2014) or reduced sensitivity to drug aversiveness (Tirelli et al., 2003; Schramm-Sapyta et al., 2009; Doremus-Fitzwater and Spear, 2016). Our experiments were conducted under low-stress conditions using mice bred on-site, and cocaine was selected due to its reliable CPP-inducing effects and relatively low aversive profile. Estrous cycles were not monitored, limiting interpretation of sex effects, especially given the known influence of estrogen and progesterone on cocaine reward (Evans and Foltin, 2010; Peart et al., 2022). Furthermore, we did not assess dose-response effects, leaving open the possibility that age-related differences in CPP may emerge at other doses. Despite these limitations, we observed significantly lower CPP in adolescent mice compared to adults, regardless of sex or the type of reward. This indicates that developmental changes in reward sensitivity may be more complex than previously assumed. A similar finding was reported by Laviola’s group, where adolescent mice, within an age range comparable to the early-to-late adolescent period examined in our study, exhibited increased impulsivity and restlessness, along with reduced or absent rewarding effects of amphetamine compared to young adult mice (> postnatal day 60) (Adriani and Laviola, 2003; Tirelli et al., 2003). While some earlier studies have reported heightened reward motivation during adolescence, many were not explicitly designed to assess reward sensitivity. In contrast, our results provide strong evidence that reward-context associations, as measured by CPP, are diminished during adolescence in specific experimental contexts, highlighting a more nuanced developmental trajectory of reward processing

Conditioned place preference is influenced by four main factors: reinforcement, which reflects the intrinsic rewarding properties of the stimulus; motivational state, encompassing the animal’s drive for reward-seeking; memory, involving the acquisition and recall of the context-reward association; and the conditioned response, representing behavioral changes following conditioning (McKendrick and Graziane, 2020). These individual contributions are difficult to disentangle within the CPP outcome, meaning that lower CPP in adolescence arises from changes in one or more of these domains. Learning impairments, however, appear unlikely. We previously demonstrated that adolescent mice develop social CPP following shorter conditioning than adults (Harda et al., 2022; Misiołek et al., 2023), and our current and past findings demonstrate robust cocaine CPP across age groups (Harda et al., 2025b), suggesting intact or even enhanced learning capacity during adolescence. Moreover, we found no evidence of increased novelty-seeking overriding conditioned responses. Although adolescents spent more time in the neutral compartment in both food and cocaine tasks, this did not co-occur with increased exploratory behavior and likely reflects baseline behavioral differences (see Supplementary Figure 4). Taken together, these findings support the interpretation that the attenuated CPP observed during adolescence is more likely due to decreased reward sensitivity or motivation rather than deficits in learning or an increase in novelty-seeking behavior.

From the perspective of behavioral learning theory, behavior is shaped through the reinforcement and punishment of actions (Vargas, 2017; Schlinger, 2021). Therefore, lower CPP in adolescents may reflect either diminished reinforcement or a reduced aversiveness of stimulus absence, or both, which together weaken associative learning. This idea aligns with neurodevelopmental research, which shows that mid-adolescence is a period of increased brain plasticity and heightened emotional reactivity (Spear, 2000; Uhlhaas et al., 2023). However, these factors alone do not fully explain the lowered reward preference we found. In humans, adolescence is marked by a stronger influence of emotional biases on decision-making and executive functions (Spear, 2013; Poon, 2017). Executive functions encompass both “cold” processes, such as logical thinking and control, and “hot” processes that involve emotions, impulsivity, and reward sensitivity, which peak during mid-adolescence (Poon, 2017). Thus, we propose that it is a developmental stage characterized by generally lower baseline reward sensitivity, unless the stimuli are highly emotional or socially salient. A similar interpretation was previously proposed by Laviola et al.’s (2003) group, who reported that during adolescence, dopamine release is lower at baseline but higher after stimulation due to a larger dopamine storage pool compared to adults (Tirelli et al., 2003). This may serve as a gating mechanism to prevent unnecessary experience-dependent plasticity, which could otherwise contribute to the emergence of psychiatric disorders (Fairchild, 2011; Galván, 2013). Understanding these developmental changes is crucial for developing strategies to prevent mental health disorders that begin in adolescence and persist into adulthood. Future research should examine how the social environment, stress, hormones, and individual behavior affect reward sensitivity and vulnerability to psychiatric disorders during this critical period. Insights from such studies could inform more effective, targeted pharmacological and psychological treatments for adolescent mental illnesses.

## Data Availability

The datasets presented in this study can be found in online repositories. The names of the repository/repositories and accession number(s) can be found in the article/Supplementary material.
