# Supplementary material for: Adolescent mice exhibit lower reward sensitivity than adults
Source: Front Behav Neurosci. 2025 Nov 3;19:1695375. doi: 10.3389/fnbeh.2025.1695375 (PMC12620450; doi:10.3389/fnbeh.2025.1695375)
Supplement: Supplementary file 1 [file Table_1.docx]

Supplementary Material

Adolescent mice exhibit lower reward sensitivity than adults

Klaudia Misiołek^1^, Magdalena Chrószcz^1^, Marta Klimczak^1^, Aleksandra Rzeszut^1^, Julia Netczuk^1^, Barbara Ziółkowska^1^, Łukasz Szumiec^1^, Maria Kaczmarczyk-Jarosz^2^, Zofia Harda^1*^, Jan Rodriguez Parkitna^1*^

^1^ Department of Molecular Neuropharmacology, Maj Institute of Pharmacology, Polish Academy of Sciences, Krakow, Poland

^2^ Department of Physiology, Maj Institute of Pharmacology, Polish Academy of Sciences, Krakow, Poland

*** Correspondence:**Corresponding Author
E-mail: Jan Rodriguez Parkitna: [janrod@if-pan.krakow.pl](mailto:janrod@if-pan.krakow.pl)
E-mail: Zofia Harda: [zofiamijakowska@gmail.com](mailto:zofiamijakowska@gmail.com)

**
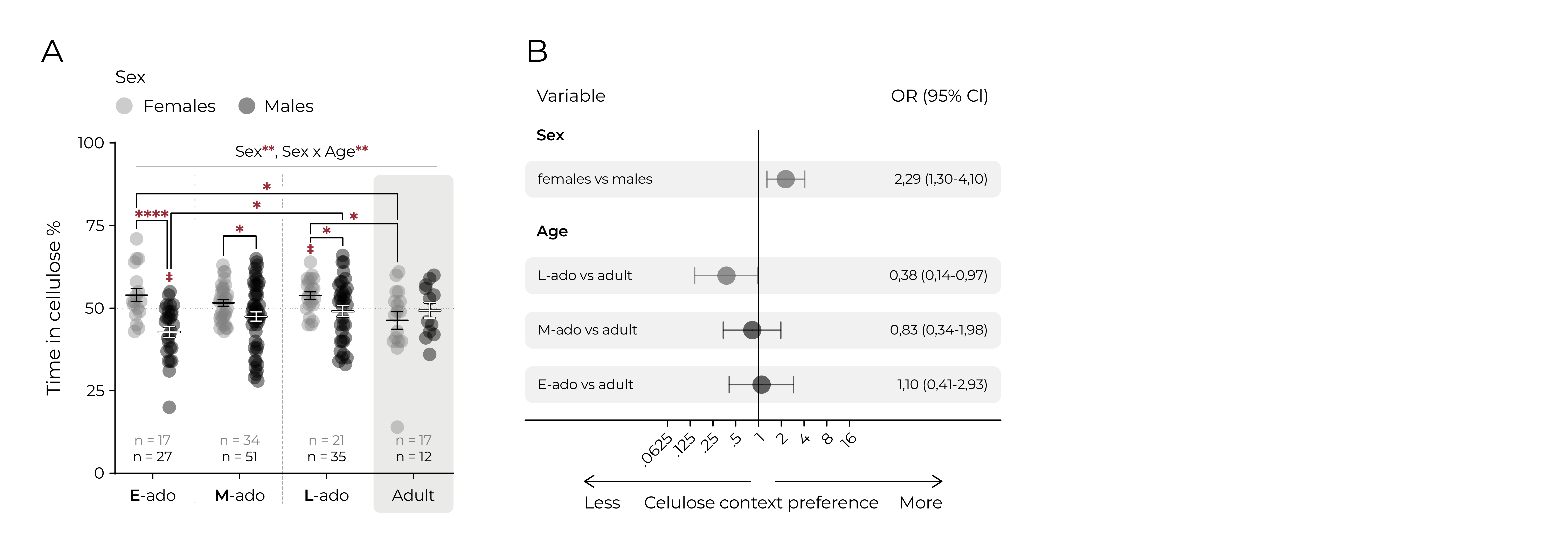
**

**Supplementary Figure 1.** Mice express significant bias in initial preference in pretest before place conditioning.

(A) Time spent in the cellulose context in pretest. Graph includes data from all animals, including animals later excluded based on criterion of initial context bias (>70% of time in any of the contexts in pretest). Each circle represents an individual animal. Whiskers represent mean and s.e.m. values. Dotted line represents random value (i.e. 50%). Females and males are shown in gray and black respectively. Statistical analysis was performed with 2-way ANOVA: Fage_(3.21)_ = 1.46, p = .230, Fsex_(1.21)_ = 10.82, p = .001, Fage*sex_(3.21)_ = 3.89, p = .009 and Tukey’s HSD, “*” corresponds to *p ≤ .05, **p ≤ .01, and ****p ≤ .0001 and “^#^”, p ≤ .08.

(B) Logistic regression estimates of the effects of sex and age on the odds of increased preference for cellulose context based on the index z-score. The circles and horizontal lines indicate the odds ratio and corresponding 95% confidence intervals. Statistically significant effects are marked gray. The odds of cellulose bedding preference in the pretest was 29% higher for females compared to males (95% CI [1.23, 4.09], p = .0046) and 37% lower for late-adolescent mice compared to adults (odds ratio 95% CI [.14, .97], p = .045).

**
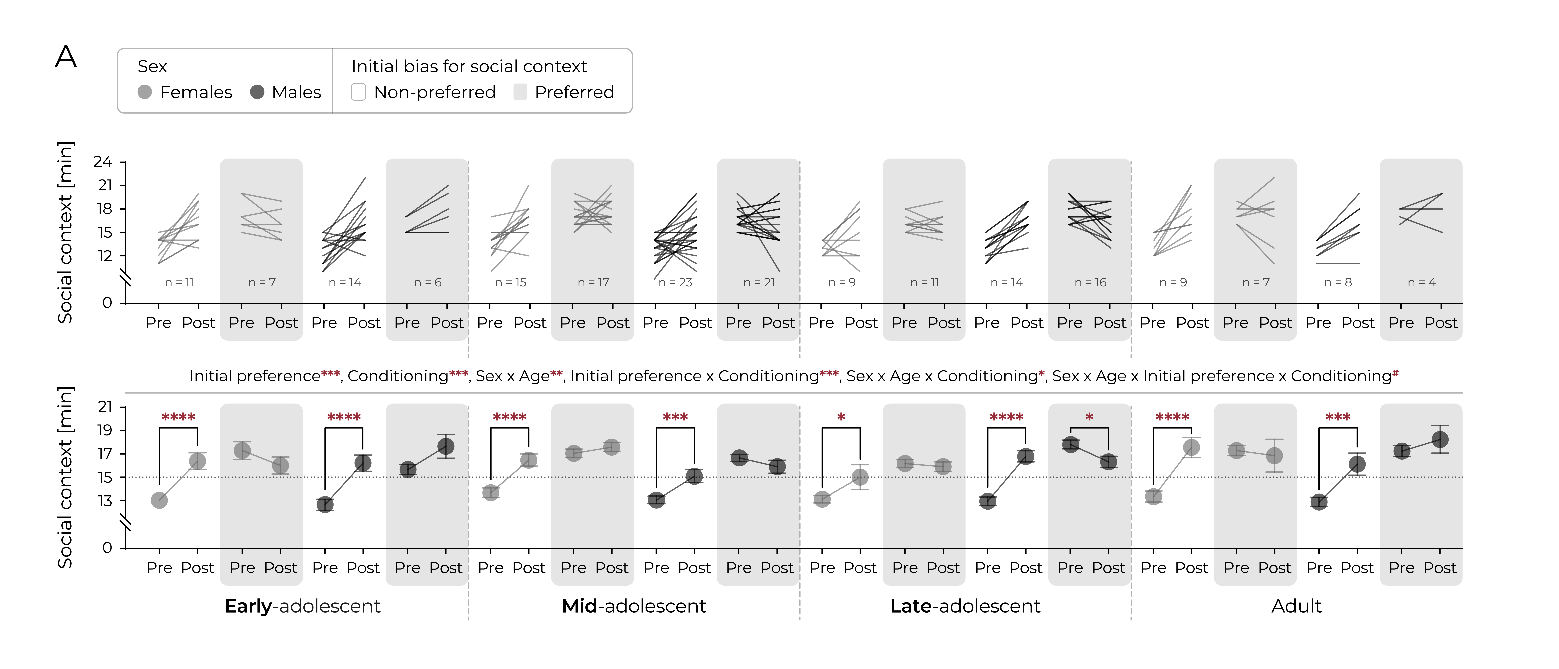
**

**Supplementary Figure 2.** Increase in place preference after conditioning differs as a function of initial bias for social context.

(A) Time spent in the social context, according to initial bias. Top panels: lines represent individual animals. Bottom panel: mean values. Circles and connecting lines represent means and matched values. Whiskers represent s.e.m. values. Dotted lines represent random value (i.e. 15 min). Females and males are shown in gray and black respectively. Females and males are shown in dark gray and black respectively. Bias for social context for initially non-preferred and preferred context are marked white and light gray background, respectively. Statistical analysis was performed using 4-way ANOVA with matched values: : Finitialbias_(4.03)_ = 95.0, p < .001, Fconditioning_(3.36)_ = 52.7, p < .001, Fsex_(4.03)_ = .49, p = .486, Fage_(4.03)_ = 1.02, p = .386, Fsex*age_(4.03)_ = 3.90, p = .010, Fsex*initialbias_(4.03)_ = 1.47, p = .227, Fage*initialbias_(4.03)_ = .04, p = .990, Fsex*conditioning_(3.36)_ = .41, p = .523, Fage*conditioning_(3.36)_ = 1.85, p = .140, Fconditioning*initialbias_(3.36)_ = 59.44, p < .001, Fsex*age*initialbias_(4.03)_ = .58, p = .630, Fsex*age*conditioning_(3.36)_ = 2.66, p = .050, Fsex*initialbias*conditioning_(3.36)_ = .06, p = .806, Fage*initialbias*conditioning_(3.36)_ = .63, p = .599, Fsex*age*initialbias*conditioning_(3.36)_ = 2.61, p = .053, and post hoc Tukey’s HSD, ‘*’ corresponds to p ≤ .05, ‘**’, p ≤ .01, ‘***’, p ≤ .001, ‘****’ p ≤ .0001.

**
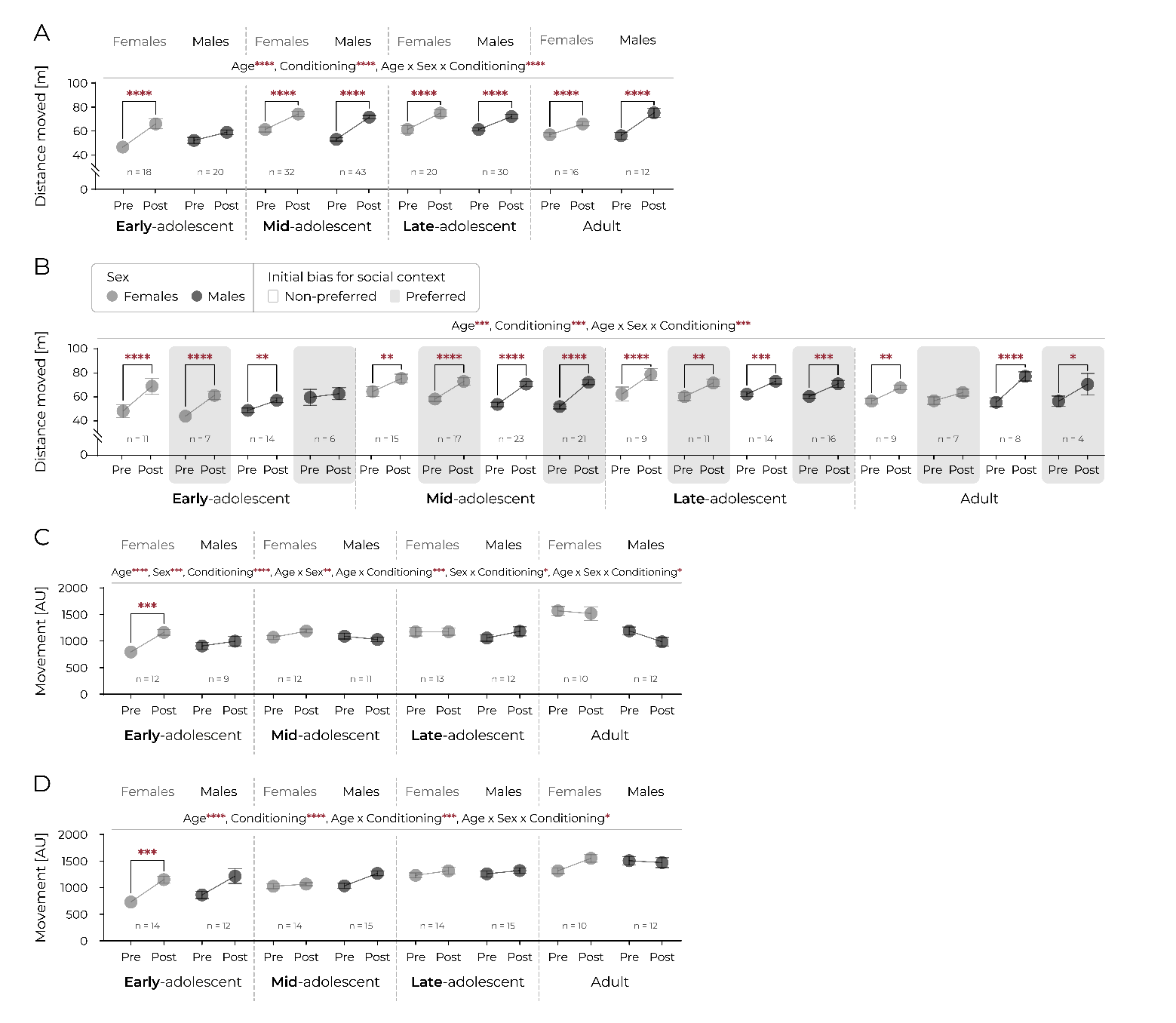
**

**Supplementary Figure 3.** Analysis of motor activity during the pre-test and posttest phases in social, cocaine, and food CPP paradigms revealed no evidence of a confounding effect on context preference.

(A) Distance moved during the pre- and post-test session in unbiased social CPP. Circles and connecting lines represent means and matched values. Whiskers represent s.e.m. values. Females and males are shown in gray and black respectively. Statistical analysis was performed using 3-way ANOVA with matched values was performed: Fconditioning_(1.18)_ = 242.4, p < .0001, Fage_(3.18)_ = 11.16, p < .0001, Fsex_(1.18)_ = .35, p = .554, Fage*sex_(3.18)_ = 1.80, p = .147, Fage*conditioning_(3.18)_ = 1.11, p = .346, Fsex*conditioning_(1.18)_ = .420, p = .994, Fage*sex*conditioning_(3.18)_ = 7.45, p < .0001 and post hoc Tukey’s HSD, ‘****’ corresponds to p ≤ .0001.

(B) Distance moved during the pre- and post-test session, according to initial bias in social CPP. Circles and connecting lines represent means and matched values. Whiskers represent s.e.m. values. Females and males are shown in dark gray and black respectively. Bias for social context for initially non-preferred and preferred context are marked white and light gray background, respectively. Statistical analysis was performed using 4-way ANOVA with matched values: Fconditioning_65.64)_ = 214.03, p < .0001, Fage_(184.40)_ = 9.62, p < .001, Fsex_(184.40)_ = .09, p = .759, Finitialbias_(184.40)_ = 1.26, p = .263, Fsex*age_(184.40)_ = 2.06, p = .111, Fsex*initialbias_(184.40)_ = 2.63, p = .107, Fsex*conditioning_(65.64)_ = .06, p = .812, Fage*conditioning_(65.64)_ = 1.17, p = .321, Fconditioning*initialbias_(65.64)_ = 1.58, p = .210, Fage*sex*conditioning_(65.64)_ = 7.06, p < .001, Fsex*initialbias*conditioning_(65.64)_ = .00, p < .953, Fage*initialbias*conditioning_(65.64)_ = 1.63, p < .183, Fage*sex*initialbias_(184.40)_ =.94, p = .420, Fage*sex*initialbias*conditioning_(65.64)_ = .22, p = .885 and post hoc Tukey’s HSD, ‘*’ corresponds to p ≤ .05, ‘**’, p ≤ .01, ‘***’, p ≤ .001, ‘****’ p ≤ .0001.

(C) Distance moved during the pre- and post-test session in cocaine CPP. Circles and connecting lines represent means and matched values. Whiskers represent s.e.m. values. Females and males are shown in gray and black respectively. Statistical analysis was performed using 3-way ANOVA with matched values was performed: Fconditioning_(1.18)_ = 3.62, p = .060, Fage_(3.83)_ = 10.78, p < .0001, Fsex_(1.18)_ = 12.33, p = .001, Fage*sex_(3.83)_ = 5.43, p = .002, Fage*conditioning_(3.82)_ = 7.69, p = .0001, Fsex*conditioning_(1.18)_ = 5.82, p = .018, Fage*sex*conditioning_(3.83)_ = 3.03, p = .034 and post hoc Tukey’s HSD, ‘*’ corresponds to p ≤ .05, ‘**’, p ≤ .01, ‘***’, p ≤ .001, ‘****’ p ≤ .0001.

(D) Distance moved during the pre- and post-test session in food CPP. Circles and connecting lines represent means and matched values. Whiskers represent s.e.m. values. Females and males are shown in gray and black respectively. Statistical analysis was performed using 3-way ANOVA with matched values was performed: Fconditioning_(1.98)_ = 39.48, p < .0001, Fage_(3.98)_ = 27.29, p < .0001, Fsex_(1.98)_ = 3.20, p = .076, Fage*sex_(3.98)_ = .33, p = .801, Fage*conditioning_(3.98)_ = 6.77, p = .0003, Fsex*conditioning_(1.98)_ = .59, p = .445, Fage*sex*conditioning_(3.98)_ = 2.83, p = .042 and post hoc Tukey’s HSD, ‘*’ corresponds to p ≤ .05, ‘***’, p ≤ .001, ‘****’ p ≤ .0001.


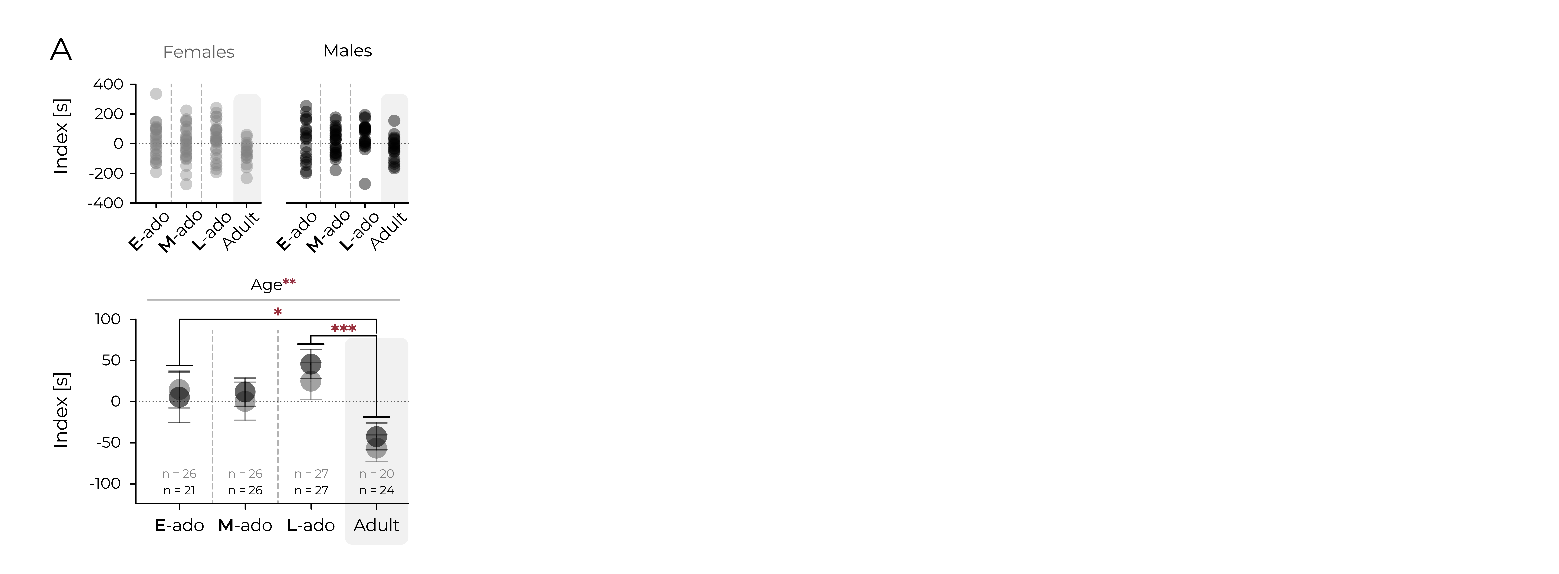


**Supplementary Figure 4.**  Adult mice, regardless of sex, spent less time in the neutral context than juvenile mice after conditioning. Related to Figures 2C and 3C.

(A) Difference in seconds between time spent in neutral context posttest and time spent in neutral context pre-test (index). Cocaine and food CPP data were combined. Top panels: individual animals. Bottom panel: mean values. Whiskers represent s.e.m. values. Dotted lines represent no change. Statistical analysis was performed using 2-way ANOVA: Fage_(3.19)_ = 5.43, p = .0001, Fsex_(1.19)_ = .37, p = .542, Fage*sex_(3.19)_ = .18, p = .907 and post hoc Tukey’s HSD, ‘*’ corresponds to p ≤ .05, ‘**’, p ≤ .01.

**Supplementary Table 1** Age, weight and number of excluded animals in all CPP tests.

| CPP test | Sex | Age bin | Excluded n^a^ | Tested n | Subject's weight at post-test [g] | | |  | Subject's age at post-test (days) | | |
| --- | --- | --- | --- | --- | --- | --- | --- | --- | --- | --- | --- |
|  |  |  |  |  | Range | Mean | SEM |  | Range | Mean | SEM |
| Social (unbiased) | Females | Early-adolescent | 1 | 18 | 4,5 | 13,9 | 0,3 |  | 2 | 31 | 0,1 |
|  |  | Mid-adolescent | 0 | 32 | 4,5 | 15,2 | 0,2 |  | 5 | 37 | 0,4 |
|  |  | Late-adolescent | 0 | 20 | 2,7 | 15,5 | 0,2 |  | 4 | 44 | 0,4 |
|  |  | Adult | 1 | 16 | 4,1 | 19,8 | 0,3 |  | 2 | 82 | 0,2 |
|  | Males | Early-adolescent | 1 | 20 | 4,2 | 17,6 | 0,3 |  | 2 | 32 | 0,1 |
|  |  | Mid-adolescent | 3 | 44 | 8,8 | 17,4 | 0,4 |  | 4 | 37 | 0,3 |
|  |  | Late-adolescent | 0 | 30 | 6,7 | 19,1 | 0,3 |  | 6 | 42 | 0,3 |
|  |  | Adult | 0 | 12 | 3,9 | 23,7 | 0,3 |  | 8 | 83 | 0,9 |
| Social (biased) | Females | Early-adolescent | - | 11 | 5,2 | 16,3 | 0,7 |  | 3 | 32 | 0,3 |
|  |  | Mid-adolescent | - | 19 | 7,4 | 17,1 | 0,6 |  | 5 | 37 | 0,5 |
|  |  | Late-adolescent | - | 17 | 7,2 | 18,3 | 0,6 |  | 6 | 42 | 0,4 |
|  |  | Adult | - | 9 | 2,9 | 20,1 | 0,3 |  | 2 | 82 | 0,2 |
|  | Males | Early-adolescent | - | 18 | 7,4 | 16,1 | 0,5 |  | 2 | 32 | 0,1 |
|  |  | Mid-adolescent | - | 24 | 11,7 | 15,6 | 0,5 |  | 4 | 36 | 0,3 |
|  |  | Late-adolescent | - | 7 | 6,3 | 18,0 | 1,0 |  | 5 | 43 | 0,6 |
|  |  | Adult | - | 8 | 3,9 | 23,6 | 0,5 |  | 8 | 83 | 1,2 |
| Cocaine | Females | Early-adolescent | 1 | 12 | 4,5 | 11,8 | 0,3 |  | 2 | 31 | 0,2 |
|  |  | Mid-adolescent | 0 | 12 | 3,3 | 14,5 | 0,3 |  | 1 | 38 | 0,1 |
|  |  | Late-adolescent | 0 | 13 | 2,0 | 14,9 | 0,2 |  | 2 | 44 | 0,2 |
|  |  | Adult | 1 | 10 | 2,6 | 20,8 | 0,2 |  | 3 | 118 | 0,4 |
|  | Males | Early-adolescent | 0 | 9 | 5,5 | 13,1 | 0,6 |  | 1 | 31 | 0,2 |
|  |  | Mid-adolescent | 0 | 11 | 2,6 | 17,9 | 0,3 |  | 3 | 38 | 0,4 |
|  |  | Late-adolescent | 0 | 12 | 5,4 | 18,8 | 0,5 |  | 1 | 43 | 0,1 |
|  |  | Adult | 2 | 12 | 7,5 | 25,5 | 0,6 |  | 6 | 112 | 0,7 |
| Palatable food | Females | Early-adolescent | 0 | 14 | 3,8 | 11,9 | 0,3 |  | 2 | 31 | 0,2 |
|  |  | Mid-adolescent | 1 | 14 | 2,5 | 14,6 | 0,2 |  | 0 | 38 | 0,0 |
|  |  | Late-adolescent | 0 | 14 | 4,1 | 16,3 | 0,3 |  | 2 | 44 | 0,2 |
|  |  | Adult | 2 | 10 | 4,6 | 20,7 | 0,4 |  | 2 | 115 | 0,3 |
|  | Males | Early-adolescent | 2 | 12 | 7,7 | 13,6 | 0,7 |  | 2 | 31 | 0,2 |
|  |  | Mid-adolescent | 0 | 15 | 4,9 | 16,4 | 0,4 |  | 0 | 38 | 0,0 |
|  |  | Late-adolescent | 0 | 15 | 2,8 | 18,9 | 0,2 |  | 1 | 45 | 0,1 |
|  |  | Adult | 0 | 12 | 6,2 | 28,1 | 0,6 |  | 1 | 102 | 0,1 |

^a^Number of mice that did not meet criterion of spending less than 70% of time in any of conditioning contexts

**Supplementary Table 2** Nutritional comparison of ingredients of palatable food mix used in food CPP test.

| Component of palatable food mix | kcal/g | Carbohydrate % | Fat % | Protein % | Added Sugar % | Sodium % |
| --- | --- | --- | --- | --- | --- | --- |
| Chow (Altromin) | 3,3 | 65,0 | 11,0 | 24,0 | - | 0,2 |
| Froot Loops | 3,9 | 87,0 | 3,8 | 5,1 | 31,0 | 1,4 |
| Cheetos | 4,8 | 62,0 | 23,0 | 6,1 | 7,6 | 3,2 |
| Oreos | 4,8 | 68,0 | 20,0 | 5,3 | 38,0 | 0,7 |
